# Validity and reliability of the Swedish version of the Visual CARE Measure for assessing children’s perceptions of nurses’ empathy

**DOI:** 10.1007/s00431-025-05979-z

**Published:** 2025-01-18

**Authors:** Angelica Wiljén, John Chaplin, Stefan Nilsson, Katarina Karlsson, Joakim Öhlén, Anneli Schwarz

**Affiliations:** 1 Institute of Health and Care Sciences, Sahlgrenska Academy, University of Gothenburg, Gothenburg, Sweden; 2 Queen Silvia Children’s Hospital, Sahlgrenska University Hospital, Gothenburg, Sweden; 3 Centre for Person-Centred Care, University of Gothenburg, Gothenburg, Sweden; 4 Faculty of Caring Science, Work Life and Social Welfare, University of Borås, Borås, Sweden; 5 Palliative Care Centre, Sahlgrenska University Hospital, Gothenburg, Sweden; 6 Södra Älvsborgs Hospital, Borås, Sweden

**Keywords:** Questionnaire, Empathy, Child, Person-centred, Chronic diseases

## Abstract

The purpose of this study was to translate and validate a questionnaire to be used by children with chronic diseases during procedures. Specific research questions were as follows: Is the translated versions reliable? Is there a correlation between VCM and another questionnaire measuring discomfort to enhance the validity of VCM? The three versions of Visual CARE measure (VCM) were translated following the principles of good practice for translation and cultural adaptation of patient-reported outcome measures, according to the International Society for Pharmacoeconomics and Outcomes Research (ISPOR). Cognitive debriefing interviews with children, parents, and healthcare professionals were carried out. The correlation between empathy and discomfort was evaluated using DISCO-RC. The final versions of VCM were administered to children and their parents after a needle procedure or a nasal tube insertion. The Cronbach’s alpha was 0.89 for the 5Q version, 0.93 for the 10Q version, and 0.96 for the 10Q-Parent version, which implies high internal consistency. The intra-class correlation was 0.623 for VCM 5Q, with a mean difference of 1.35 and an ICC of 0.767 for VCM 10Q, with a mean difference of 1.17. This is considered a good agreement between children’s and their parents’ assessments. A significant difference in the children’s reports could be seen when experiencing high discomfort as opposed to low discomfort.

*Conclusion*: VCM is a useful Patient-Reported Experience Measure (PREM) that can help enhance the quality of care for children in a paediatric setting by capturing their perception of the empathy shown.

**Table:** 

**What is known:** *• Parents’ by-proxy reports are not always aligned with children’s self-reports. In healthcare situations, children can be hurt when they are ignored.* *• Research shows that empathic encounters relieve distress, improve the relationship between the patient (who could be a child) and carer, and create an open, trusting relationship.* **What is new:** *• This study shows that all three versions of the Visual CARE Measure are valid for assessing children’s experience of empathy shown by nurses during a procedure..* *• This is one of the first studies that confirms the correlation between children’s experience of nurses’ levels of empathy and the child’s discomfort.* *• This study shows that the Visual CARE Measure can support the evaluation of children’s rights with regards to communication in a clinical context.*

**Supplementary Information:**

The online version contains supplementary material available at 10.1007/s00431-025-05979-z.

## Introduction

Research shows that children can be hurt when their wishes are neglected, or no one listens to them in health care situations [1]. This predicament is more frequent for children with chronic diseases due to their recurring contact with healthcare [2]. Healthcare professionals are at risk of forgetting empathy during care procedures because completing the procedure becomes the focus [3]. Research indicates that proxy-reports do not consistently align with the self-reports of children [4]. To establish satisfactory quality of care and to ensure patients are heard, patient reported experience measures (PREM) are needed [5, 6]. In a systematic review on paediatric PREMs [7], only 13.4% of the PREMs were to be completed by the child. In addition, less than 50% of paediatric departments in Sweden used questionnaires that target children [8].

Children experience physical and mental discomfort in clinical procedures such as anxiousness during venipuncture [9]. A first step for healthcare professionals such as nurses to alleviate distress and physical or mental suffering in patients is to accept, recognize and understand their suffering. Empathy supports healthcare professionals in recognizing the patients suffering [10].

There is a new standard, iSUPPORT (International collaborative rights-based standards to SUpport Paediatric Patients during clinical prOcedures by Reducing harm and establishing Trust) that is based on children’s rights when a child undergoes a healthcare procedure. This standard highlights the importance of creating trust and minimizing anxiety and distress through the healthcare professionals’ approach, communication and listening [11]. Children’s rights are protected by the agreement set out by The United Nations convention on the rights of the child. The agreement explains what responsibilities the government has, who is a child and what rights a child has. These rights include that adults should take the child’s opinions seriously and listen to them, and that the child gets information in a language that he or she understands [12].

There is a shortage of instruments measuring empathy in paediatric care and none in Swedish [13]. We argue that an effective questionnaire should be concise, child friendly and freely accessible. According to these criteria, the authors identified the Visual CARE Measure (VCM) as a suitable PREM questionnaire. The purpose of this study was to translate and validate a questionnaire for children with chronic diseases during procedures. Specific research questions were: Is the translated versions reliable? Is there a correlation between VCM and another questionnaire measuring discomfort supporting the validity of VCM?

## Method

### Design and settings

The study was performed in two steps, first involving translation, and then validation. The first step followed principles of good practice for translation and cultural adaptation of Patient-Reported Outcome Measures, [14]. The second step included a validation process consisting of face validity, convergent validity, inter-rater reliability, internal consistency, explorative factor analysis and descriptive statistics for the three translated versions of VCM. The initial translation process was carried out in one hospital in southwest Sweden during 2019, with the validation process being carried out from 2020 to 2024 in three different hospitals in southwest Sweden and in different locations, such as in-patient and out-patient care.

### The instrument

The VCM instrument is a 5–10 item self-rating questionnaire with a five-step Likert rating scale based on facial expressions ranging from “poor” or “not very good” to “excellent.” It is possible to use “does not apply.” The language is adapted, and images are used to support the text [15]. VCM evolved from the Consultation and Relational Empathy CARE Measure (CARE measure), which is a validated instrument for adults [16, 17]. VCM has previously been validated in two other contexts [15, 18]. Three different versions are used, depending on the age and ability of the child: VCM 5Q with 5 items for children aged 7–11 years, VCM 10Q with 10 items for children aged 12–17 years and VCM 10Q-parent, i.e., a by-proxy version for parents of children aged 0–6 years or children who cannot answer the questionnaire themselves due to a disability. If an answer was in between scale steps or two answers were chosen, the first and last author let chance decide the outcome by drawing a lot of either the highest or lowest number. Up to two missing values or the use of “does not apply” are allowed in the 10-item versions and should be replaced by the average score. If more than two answers were missing, the questionnaire was excluded. In the 5-item VCM, only one missing item or ‘does not apply’ was allowed and replaced by the average score. The scale scores range from 10–50 for the 10-item versions and from 5 to 25 for the 5-item version.

### Translation process

The translation process of the three versions of VCM, see supplementary files, comprised permission from the developer, a forward translation, a back-translation and a cognitive debriefing. In the cognitive debriefing, children of the correct age for each version were interviewed using a “think aloud” method whereby the child talks freely about each question and word in the questionnaire: The healthcare professionals participated in focus groups with a “think aloud” method for both VCM 5Q and VCM 10Q. The parents participated in focus groups with a “think aloud” method for VCM 10Q-Parent. If changes were made at this stage, the cognitive debriefing was done a second time in interviews with a “think aloud” method.

### Recruitment and data collection for the validation

A total of 690 envelopes were printed and coded: 225 for children aged 7–11 years, 240 for children aged 12–17 years (120 for 12–14 and 120 for 15–17) and 225 for parent of children aged of 0–6 years. All hospitals had a code list to track missing data. A total sample size of 100 per version was deemed suitable, because VCM is a 5–10 item questionnaire and over the years ten participants per item has been found to be suitable for validation purpose [19, 20].

Questionnaires were distributed by nurses, nurse assistants, and a researcher. To ensure anonymity, envelopes were sealed by the participant before being submitted. Eligibility criteria for participation were children in the age range 0–17 with a diagnosis of a chronic disease and their parents, ability to understand spoken and written Swedish, no previous exposure to the questionnaire. The questionnaires were distributed after a needle procedure or a nasal tube insertion; see Table 1 for the characteristics of the participants in the validation step. Participation could be declined without further explanation.

**Table 1:** Characteristics of participants

Characteristics	Total number (%)
Participants	310
Total	
0–6 years	103 (33)
7–11 years	100 (32)
12–17 years*	107 (35)
Gender	
Female	164 (53)
Male	146 (47)
Diagnosis**	
Mental/ Behavioural	7 (2)
Cancer/Blood	78 (25)
Infection	0 (0)
Respiratory	23 (8)
Met/Ren/Dig/End/GU^1^	112 (36)
Musc/Skin^2^	16 (5)
Neurological/sensory	21 (7)
Cardiac conditions	6 (2)
Non-specific chronic condition	47 (15)
Number of visits/years	
0–3	65 (21)
3–6	76 (24)
> 6	161 (52)
Non-specified	8 (3)
Procedure	
Needle	280 (91)
Nasal tube	10 (3)
Combination	7 (2)
Not declared	13 (4)

*In the age group 12–17 years, 53 children were between 12–14 and 54 children were 15–17

**Hardelid et al. 2014 [21]

^1^Metabolic/Renal/Digestive/Endocrine/Genitourinary

^2^Musculoskeletal/Skin

### Data analysis

#### Reliability

Inter-rater reliability was calculated as the difference between the children’s and their parents’ answers in the VCM using median, confidence interval (Cl), Wilcoxon signed rank test, and intraclass correlation coefficient (ICC). Shrout-Fleiss reliability ICC, a two-way random-effects model that treats both subjects and raters as random effects, was also used. This model accounts for the variability between subjects (different children) and variability between raters (children and their parents). Values close to 1 indicate strong consistency across raters [22].

Internal consistency was analysed with Cronbach’s alpha. Cronbach’s alpha values of > 0.70 were considered acceptable [22].

#### Validity

Face validity was assessed by asking the participants for their views and using counts of missing responses, as per Mercer et al. (2005) [16]. The relevance of VCM was assessed using counts of completed questionnaires, as well as missing items and incomplete questionnaires.

An exploratory factor analysis was performed to support the validity of the translated versions and demonstrate that the items still functioned as one factor. Prior to performing the exploratory factor analysis, Kaiser–Meyer–Olkin (KMO) measure of sample adequacy was calculated for suitability. Bartlett’s test of sphericity was used to test whether there was a correlation between the variables. The KMO should be > 0.5 and Bartlett’s test should be highly significant.

Convergent validity was established by calculating the Spearman correlation coefficient between VCM and DISCOmfort in Research with Children (DISCO-RC) [23]. DISCO-RC is a questionnaire for self-report of children’s discomfort during medical research procedures. No golden standard was found, therefore a questionnaire focusing on children’s experience during medical procedures was chosen. When testing convergent validity, a strong negative correlation to DISCO-RC was expected, because the scales are at opposing ends. For more information about DISCO-RC see supplementary files.

Item-convergent validity was assessed by calculating the correlation between each item and its own scale.

#### Descriptive statistics

For the group comparisons, the total VCM score was described with mean and standard deviation. For the ceiling and floor effect, the total VCM score was described with median, range, and mode. Ceiling effect, where the range of responses is at the upper end of the scale, and floor effect, where most of the responses are at the lowest possible end of the scale were looked at to determine the validity and reliability of the newly translated instrument.

If the child used one of the two highest options in any of the items in DISCO-RC the child was considered as experiencing high discomfort. A comparison of the answers in VCM was made between children with high vs low discomfort by calculating the mean difference using Fisher’s non-parametric permutation test, with 95% CI.

All significant tests were two sided and were conducted at 5% significance level. All statistical analyses were performed using IBM SPSS, version 29 (IBM Corp, Armonk, NY).

## Results

### Translation

The translated versions were adapted based on content analysis from 10 healthcare professionals, 9 children, and 7 parents in the first cognitive debriefing, and 6 children, 2 parents, and 2 healthcare professionals in the second one. The participants struggled with some items, for example, item 4 in the VCM 5Q (Explaining things, answering questions, giving you clear information and instructions). One child said: “I don’t know what information or instructions are.” In the 10Q versions, the older children and the parents struggled with item 9 (Helping you to take control, exploring with you what you can do to improve your health yourself) or item 10 (Making a plan of action, discussing the options, involving you as much as you want). One child said: “I don’t understand that question” and another asked “Take control of what?”.

### Reliability

A subsample of 83 VCM 5Q and 92 VCM 10Q were used to compare the agreement between children and their parents VCM 10Q-Parent. The ICC was 0.623 for VCM 5Q, with a mean difference of 1.35 and an ICC of 0.767 for VCM 10Q, with a mean difference of 1.17, as seen in Table 2. This is considered a good agreement between children and parents, even though children in general scored lower than their parents.

**Table 2:** ICC for children aged 7–11 and 12–17 compared with their parents. The five items in the VCM-10Q-Parent that are in concordance with the VCM 5Q (Item 1,2,3,8,10) were used to compare the younger children’s five-item version with their parents’ ten-item version

			Difference Parents – Children		Intraclass Correlation Coefficient (ICC)
Variable	Parents Mean (95% CI Limits of Agreement) (SD) Median (Min; Max) *n* =	Children Mean (95% CI Limits of Agreement) (SD) Median (Min; Max) *n* =	Mean (95% CI Limits of Agreement) (SD) Median (Min; Max) *n* =	Systematic changes *p*-value	Shrout-Fleiss reliability: random set
Total score VCM 5Q (7–11 years)	22.5 (16.0; 29.0) (3.3) 24.0 (11.0; 25.0) *n* = 83	21.1 (13.2; 29.1) (4.0) 23.0 (6.0; 25.0) *n* = 83	1.35 (−4.61; 7.31) (3.04) 1.00 (−5.00; 11.00) *n* = 83	0.0001	0.623
Total score VCM 10Q (12–17 years)	43.9 (30.9; 56.8) (6.6) 45.0 (10.0; 50.0) *n* = 92	42.8 (28.5; 57.1) (7.3) 44.5 (10.0; 50.0) *n* = 92	1.07 (−7.80; 9.93) (4.52) 0.00 (−13.00; 15.00) *n* = 92	0.0135	0.782

Internal consistency of VCM 5Q was analysed from 100 questionnaires. Cronbach’s alpha was 0.89. VCM 10Q was analyzed from 107 questionnaires. Cronbach’s alpha was 0.94. VCM 10Q-Parent was analyzed by 103 questionnaires. Cronbach’s alpha was 0.96.

### Validity

**Fig. 1:**
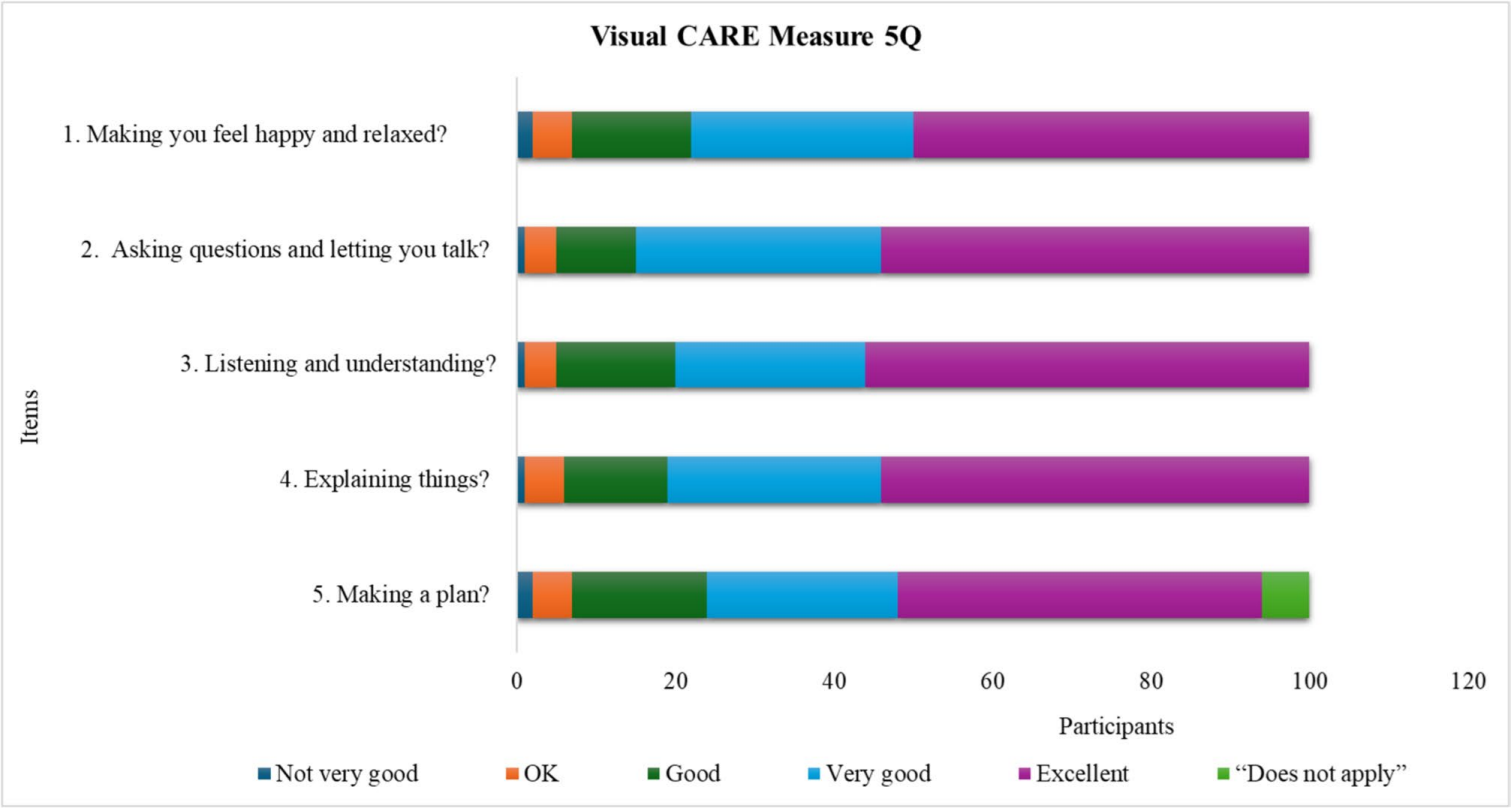
Response rates for the different items in the Visual CARE Measure (VCM) 5Q: 100 participants. The figure shows each item in VCM 5Q and how many participants used the different response options for each item. The colours show the total number of participants who chose one of the different alternatives

**Fig. 2:**
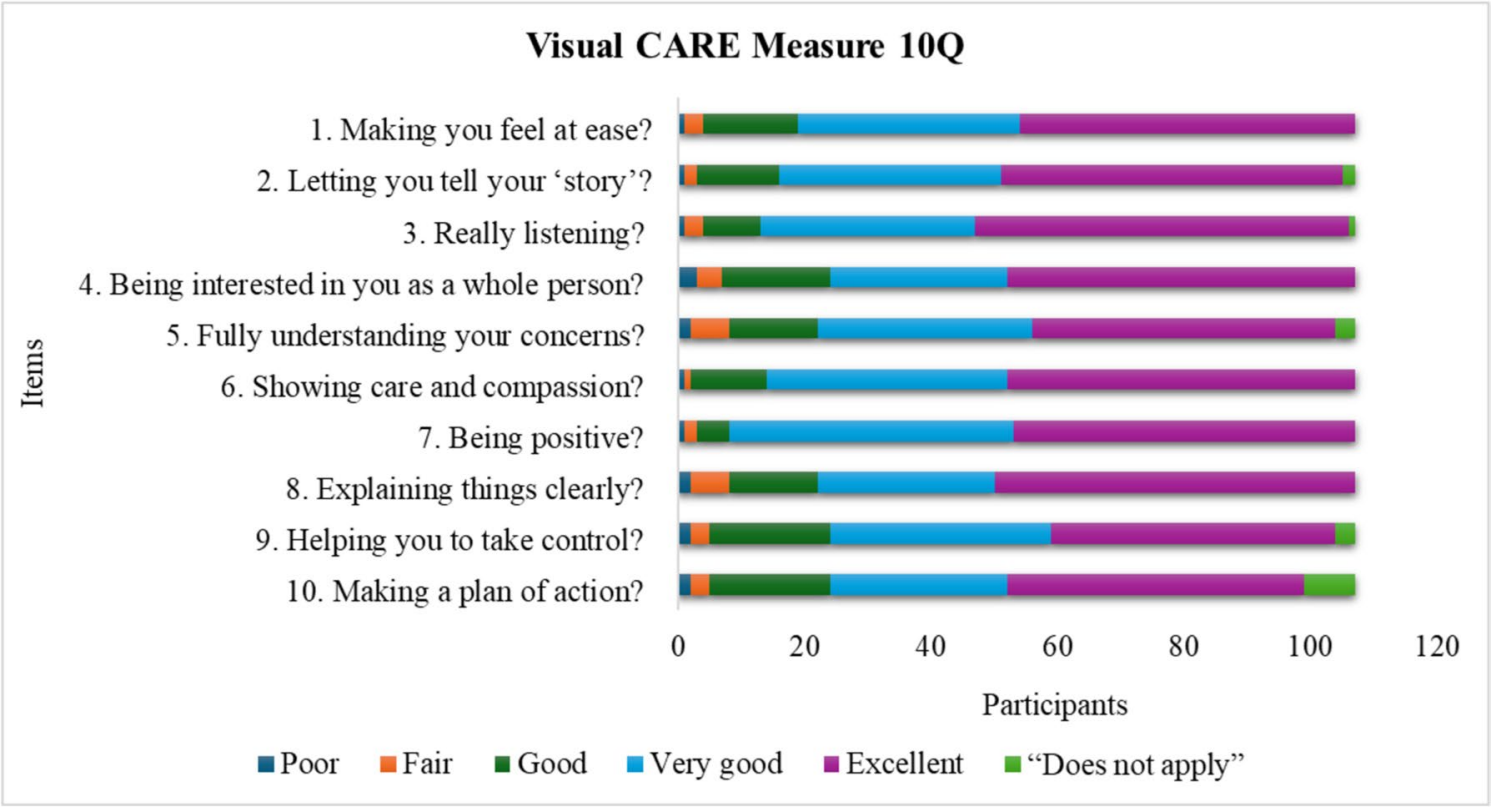
Response rates for the different items in Visual CARE Measure (VCM) 10Q: 107 participants. The figure shows each item in the VCM 10Q and how the participants responded. The colours show the total number of participants who chose one of the different alternatives

**Fig. 3:**
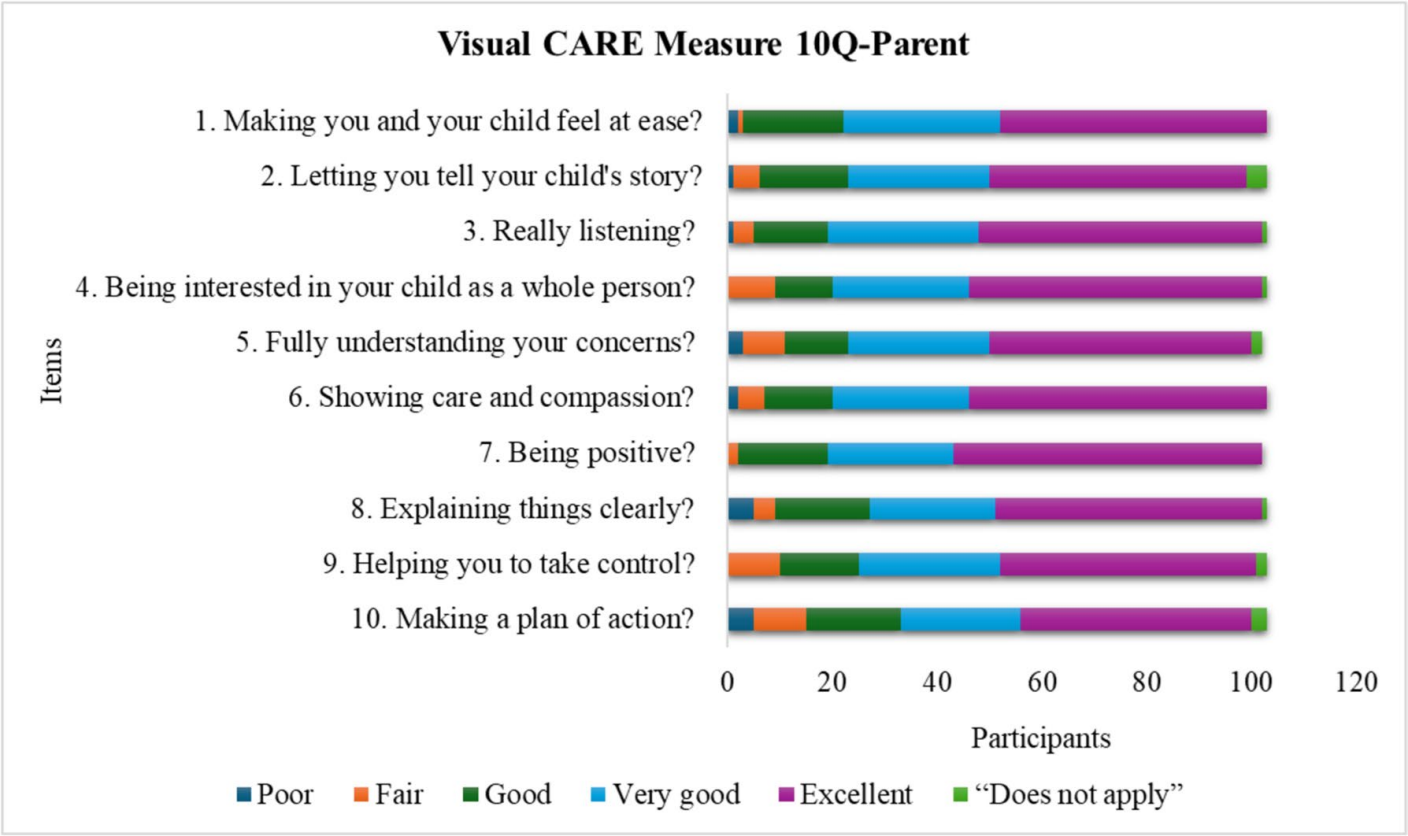
Response rates for the different items in Visual CARE Measure (VCM) 10Q-Parents: 103 participants. The figure shows each item in the VCM 10Q-Parent and how the participants responded. The colours show the total number of participants who chose one of the different alternatives

The total number of participants answering all items was 310 out of 371 (84%) (for children 207/239 [87%] and for parents 103/132 [78%]). A total of 30 (8%) questionnaires were incomplete. The most frequent reason for incomplete questionnaires was lack of responses on the reversed side. The number of children selecting the option “does not apply” was 31 (13%) children. The item for which most children used “does not apply” were item 5 in the VCM 5Q and item 10 in the VCM 10Q, as shown in Figs. 1 and 2. Eighteen (14%) parents selected the “does not apply” option and this was mostly used for items 2 and 10, as shown in Fig. 3.

The KMO was 0.87 for VCM 5Q, 0.94 for VCM 10Q, and 0.94 for VCM 10Q-Parent. The scree plot (supplementary materials) was used to choose the number of latent factors (subscales) and shows there is one factor explaining more than 65% of the variation in the data.

Convergent validity was demonstrated through significantly negative correlations between VCM 5Q and DISCO-RC (*r* = −0.30, *p* = 0.004) based on 91 samples. It was also demonstrated in VCM 10Q and DISCO-RC (*r* = −0.41, *p* < 0.001) based on 102 samples.

All items correlated significantly with their own scale, indicating good item-convergent validity. The correlation between each item and its own scale corrected for overlap ranged from 0.60–0.78 in the VCM 5Q, between 0.71 and 0.84 in the VCM 10Q and between 0.74 and 0.89 in the VCM 10Q- Parent.

### Descriptive statistics

A total of 251 envelopes were missing, 81/225 of VCM 5Q, 103/240 of VCM 10Q, and 67/225 of VCM 10Q-Parent. A ceiling effect (score = 25 for VCM 5Q, and score = 50 for VCM10Q, VCM 10Q-Parent) was noted in all versions. A total of 26% of VCM 5Q, 23% of VCM 10Q, and 30% of VCM 10Q-Parent reached maximum score. No significant floor effect (score = 5 for VCM 5Q and score = 10 for VCM10Q, VCM 10Q-Parent) was noted. The total score ranged from 6 to 25 in the 5-item version for children in the age range 7–11, with a median of 23 and a mode of 25. The total score ranged from 10 to 50 in the 10-item version for children in the age range 12–17, with a median of 44 and a mode of 50. The total score for the 10-item version for the parents ranged from 17–50, with a median of 45 and a mode of 50. For the response rate of the specific items, see Figs. 1, 2, and 3.

A Fisher´s non-parametric test showed a statistically significant difference between the groups with high levels of discomfort and the group with low levels of discomfort in terms of empathy for both VCM 5Q (*p* = 0.011) and VCM 10Q (*p* = 0.005), as shown in Table 3.

**Table 3:** Comparison between children with high or low levels of discomfort as seen in DISCO-RC in the age range 711 (VCM 5Q) and 12–17 (VCM 10Q) and their reports on nurses’ levels of empathy, as seen in the total VCM score

Variable	High levels of discomfort (*n* = 36)	Low levels of discomfort (*n* = 56)	*p*-value	Difference between groups Mean (95% CI)
Total score VCM 5Q	19.9 (4.7) 21 (6; 25) (17.5; 23) (18.3; 21.5) *n* = 36	22.1 (3.3) 23 (12; 25) (20; 25) (21.2; 23.0) *n* = 56	0.011	−2.24 (−3.89; −0.55)
Total score VCM 10Q	38.9 (8.3) 41 (10; 50) (35; 44) (35.7; 42.0) *n* = 29	43.8 (6.7) 46 (21; 50) (40; 50) (42.2; 45.4) *n* = 73	0.0050	−4.93 (−8.00; −1.67)

## Discussion

This is the first time that VCM has been translated and validated to be used in Swedish paediatric care. By including different participants during the translation process, we received acceptable versions of VCM with some cultural adaptations. The translation process showed that parents and healthcare professionals found the VCM to be valuable in a general paediatric setting. Even though we did not ask children themselves about their view of empathic communication, the literature shows that it is valued by children with chronic diseases [24]. A limitation is that we cannot verify that the participants only evaluated the encounter during the specific procedure and did not evaluate earlier encounters. We tried to minimize this by handing out the questionnaires in proximity of the procedure and by informing the participants that they should think about the encounter during one specific procedure. Social desirability is a potential bias in our study, as the nurse who asked the participants to participate could have been the same nurse who participated in the procedure. However, the children and parents were asked to fill out the questionnaire privately in a room without any healthcare professionals present and they sealed their envelopes before returning them.

Previous research has shown an inconsistency between the experiences of children and their parents regarding the children’s health [4]. In this study, we found a consistency in parent and children’s responses. This could be explained by both being present during the procedure, which might have made it easier for the parent to interpret the child’s perception.

Nevertheless, we might ask: is one instrument enough to validate nurses’ levels of empathy? Previous research about empathy in nursing has made use of self-reports but according to a literature review of Nembhard et al. 2023 [25], in order to strengthen the field of such research, controlled studies and other person measures of empathy should be included.

Since healthcare professionals’ self-reports regarding their empathy do not accurately reflect the patients’ experience, measures tailored to the patients’ perspective are important [26, 27]. VCM focuses on empathy with questions followed by a subheading, such as “Really listening: paying close attention to what you are saying,” “Explaining things clearly: fully answering your questions, giving you enough information” and “Making a plan of action: discussing the options, involving you as much as you want.” These important items are clearly encompassed in iSUPPORT and in the UN convention on the Rights of the Child [11, 12]. VCM includes children of all ages, with or without disabilities [15], which arguably makes it a valuable instrument measuring one important factor in improving children’s rights.

This is one of the first studies to confirm the correlation between children’s experience of nurses’ levels of empathy and the child’s discomfort. Children in this study who experienced high discomfort during the procedure had a lower total score for VCM and slightly higher variation in answers compared to children who reported low levels of discomfort. This can be interpreted in different ways. For example, children expressing high discomfort may lead to nurses focusing more on the specific procedure instead of on the child’s needs or, if a child experiences discomfort during a procedure, they will project this more in the encounter. When a procedure proceeds without any cause for discomfort, the nurse finds it easier to reflect on their interaction with the child and is more likely to respond to the child’s signs of distress. We know that patients tend to rate high on Likert scales, causing a ceiling effect in PREMs. Attempts have been made to limit this ceiling effect but to no avail [28].

## Conclusion

This study shows that VCM could support children in expressing their perception of empathy during a care procedure. This perspective can provide feedback to nurses working in paediatrics to enhance their awareness of how children and their families perceive communication and care given. A more emphatic nurse helps in creating a safe and trusting relationship, which enables a child to be more involved in their care and enhances the possibility of considering children’s rights. Further research may investigate more long-term settings, such as a hospital stay, home care or outpatient care, and explore whether the instrument works as an evaluation of nurses’ levels of empathy in these settings. For future research non-Swedish-speaking participants could be included for validating the usability of the instrument in this context as this study lacked data from this group. The translated and culturally adapted Swedish versions of VCM will be freely available for both practice and research use.

## Supplementary Information

Below is the link to the electronic supplementary material.

Supplementary file1 (PDF 260 KB)

Supplementary file2 (PDF 75 KB)

Supplementary file3 (PDF 82 KB)

Supplementary file4 (PDF 372 KB)

Supplementary file5 (PDF 370 KB)

Supplementary file6 (PDF 227 KB)

## Data Availability

No datasets were generated or analyzed during the current study.

## Abbreviations

CARE Measure: The Consultation and Relational Empathy CARE Measure
DISCO-RC: DISCOmfort in Research with Children
ICC: Intraclass correlation coefficient
ISPOR: International Society for Pharmacoeconomics and Outcomes Research
Isupport: International collaborative rights-based standards to Support Paediatric Patients during clinical prOcedures by Reducing harm and establishing Trust
KMO: The Kaiser–Meyer–Olkin
PCC: Person-centred care
PREM: Patient-reported experience measures
VCM: Visual CARE Measure
