# Supplementary material for: Validity and reliability of the Swedish version of the Visual CARE Measure for assessing children’s perceptions of nurses’ empathy
Source: Eur J Pediatr. 2025 Jan 18;184(2):145. doi: 10.1007/s00431-025-05979-z (PMC11742902; doi:10.1007/s00431-025-05979-z)
Supplement: Supplementary file 2 — Supplementary file2 (PDF 75 KB) [file 431_2025_5979_MOESM2_ESM.pdf]

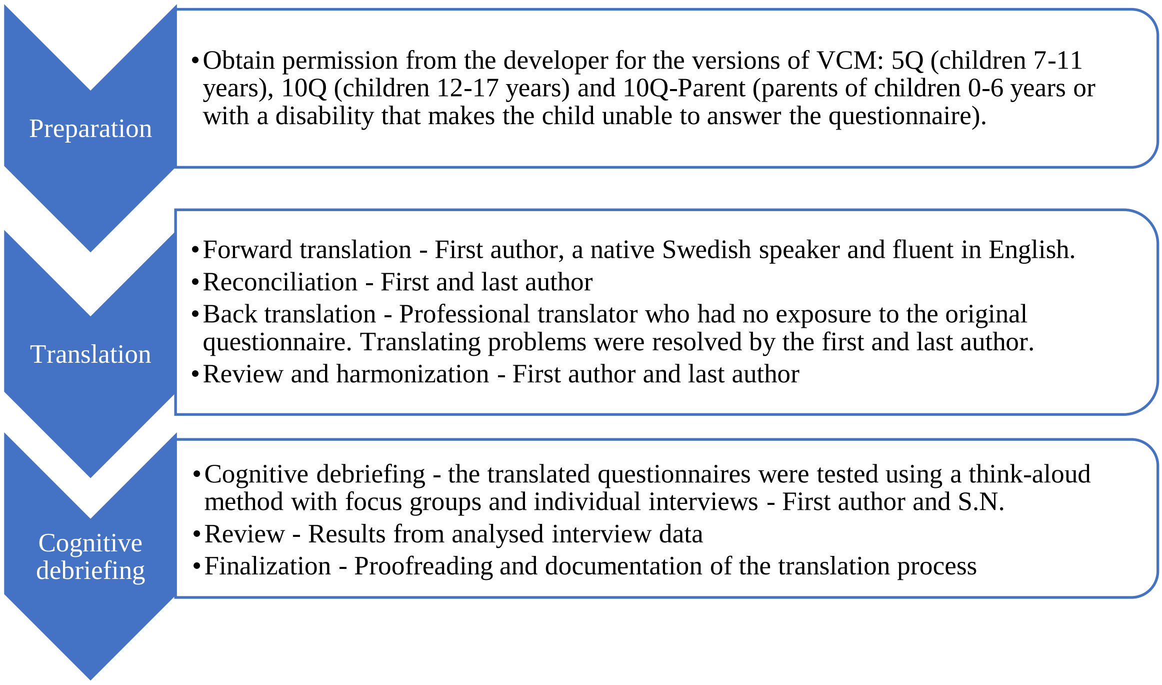

Fig. 1 Flowchart of the translation and cultural adaptation process

The figure shows the different phases of the translation and cultural adaptation process, clearly stating who did what.
