# Supplementary material for: Validity and reliability of the Swedish version of the Visual CARE Measure for assessing children’s perceptions of nurses’ empathy
Source: Eur J Pediatr. 2025 Jan 18;184(2):145. doi: 10.1007/s00431-025-05979-z (PMC11742902; doi:10.1007/s00431-025-05979-z)
Supplement: Supplementary file 3 — Supplementary file3 (PDF 82 KB) [file 431_2025_5979_MOESM3_ESM.pdf]

The validity and reliability of a Swedish version of the Visual CARE Measure to measure children's reports on nurses' levels of empathy, European Journal of Pediatrics, Wiljén, A., Chaplin, J., Nilsson, S., Karlsson, K., Öhlén, J. & Schwarz, A. Corresponding author: Angelica Wiljén, email: angelica.wiljen@gu.se

DISCOmfort in Research with Children (DISCO-RC)

DISCOmfort in Research with Children (DISCO-RC) was developed by Staphorst et al. (2015) as a questionnaire for self-report of children's discomfort during medical research procedures. The DISCO-RC version used in this study was adapted by the authors (with permission from the developer) to support the text by adding a thermometer scale to the left and adding images collected from www.bildstod.se. After adaptations, the DISCO-RC in this study was a six-item self-rating questionnaire with a five-point, colour-based Likert scale (see supplementary materials). Incomplete questionnaires were excluded.

DISCO-RC was translated into Swedish following the same guidelines, including permission from the developer, using a forward translation, back-translation and finally being tested by a group of experts.

The translated version of DISCO-RC was adjusted using interviews with 5 researchers, 2 parents and 2 older children. Adjustments to the original questionnaire were made by adding images to each item, as well as only using the 6 initial items ("Were you nervous?", "Was it annoying?", "Were you frightened?", "Was it painful?", "Were you bored?", and "Was it tiring?".

Reference

Staphorst MS, Hunfeld JA, van de Vathorst S, Passchier J, van Goudoever JB. Children's self reported discomforts as participants in clinical research. Soc Sci Med. 2015 Oct;142:154-62. eng. Epub 2015/08/28. doi:10.1016/j.socscimed.2015.08.019. Cited in: Pubmed; PMID 26310591.
